# Pharmacological Estrogen Administration Causes a FSH-Independent Osteo-Anabolic Effect Requiring ER Alpha in Osteoblasts

**DOI:** 10.1371/journal.pone.0050301

**Published:** 2012-11-27

**Authors:** Sebastian Seitz, Johannes Keller, Arndt F. Schilling, Anke Jeschke, Robert P. Marshall, Brenda D. Stride, Tim Wintermantel, Frank T. Beil, Michael Amling, Günther Schütz, Jan Tuckermann, Thorsten Schinke

**Affiliations:** 1 Department of Osteology and Biomechanics, University Medical Center Hamburg-Eppendorf, Hamburg, Germany; 2 Department of Plastic and Hand Surgery, Technische Universität München, Munich, Germany; 3 Division Molecular Biology of the Cell I, German Cancer Research Center, Heidelberg, Germany; 4 Leibniz Institute for Age Research-Fritz Lipmann Institute, Jena, Germany; Baylor College of Medicine, United States of America

## Abstract

Postmenopausal osteoporosis is characterized by declining estrogen levels, and estrogen replacement therapy has been proven beneficial for preventing bone loss in affected women. While the physiological functions of estrogen in bone, primarily the inhibition of bone resorption, have been studied extensively, the effects of pharmacological estrogen administration are still poorly characterized. Since elevated levels of follicle-stimulating hormone (FSH) have been suggested to be involved in postmenopausal bone loss, we investigated whether the skeletal response to pharmacological estrogen administration is mediated in a FSH-dependent manner. Therefore, we treated wildtype and FSHβ-deficicent (*Fshb^−/−^)* mice with estrogen for 4 weeks and subsequently analyzed their skeletal phenotype. Here we observed that estrogen treatment resulted in a significant increase of trabecular and cortical bone mass in both, wildtype and *Fshb^−/−^* mice. Unexpectedly, this FSH-independent pharmacological effect of estrogen was not caused by influencing bone resorption, but primarily by increasing bone formation. To understand the cellular and molecular nature of this osteo-anabolic effect we next administered estrogen to mouse models carrying cell specific mutant alleles of the estrogen receptor alpha (ERα). Here we found that the response to pharmacological estrogen administration was not affected by ERα inactivation in osteoclasts, while it was blunted in mice lacking the ERα in osteoblasts or in mice carrying a mutant ERα incapable of DNA binding. Taken together, our findings reveal a previously unknown osteo-anabolic effect of pharmacological estrogen administration, which is independent of FSH and requires DNA-binding of ERα in osteoblasts.

## Introduction

Osteoporosis results from an imbalance between bone formation and bone resorption, thereby causing bone loss and increased fracture risk [1]. One of the major risk factors of osteoporosis in women is menopause, when ovarian atrophy results in a decline of serum estrogen levels and increased bone resorption [2]. However, the cellular and molecular mechanisms of estrogen action are still insufficiently understood, and different mechanisms of action have been proposed to explain, how estrogen mediates its conserving effect on bone mass [3]. Increasing evidence suggested that physiological levels of estrogen prevent excessive bone resorption directly by regulating the life span of osteoclasts, which seem to require in part the presence of ERα in osteoclasts [4], [5]. However, since ovarian failure is accompanied by an increase in pituitary-derived hormones, it was alternatively proposed that FSH (follicle-stimulating hormone), and not necessarily estrogen itself, is involved in hypogonadism-induced bone loss [6], [7]. This hypothesis was supported by the findings that hypogonadic mice lacking either the FSH receptor (*Fshr^−/−^*) or the FSH ß-subunit (*Fshb^−/−^*) failed to develop the expected osteopenia, and that FSH administration activated osteoclast differentiation *in vitro* [8].

Although an inhibition of bone resorption is generally considered as the primary physiological function of endogenous estrogen in bone remodeling, it is surprising that the effects of estrogen administration at pharmacological doses are less well characterized, and there are several questions that have not been addressed to date. First, although estrogen supplementation at pharmacologic doses has been shown to positively affect bone mineral density and reduce fracture risk in postmenopausal women [9], it remained questionable whether this is i) mediated in a FSH-dependent manner and ii) soley attributable to an inhibitory effect on bone resorption. Second, while it has been established that estrogen receptor alpha (ERα) is the relevant estrogen receptor in adult bone tissue [10], it remained unanswered, which target cell mediates the osteoanabolic effect of estrogen administration at pharmacological doses. Third, although ERα is considered a dimeric nuclear receptor functioning as a transcription factor, several studies have reported ERα can be associated with the cell surface, where it mediates rapid biological signals independent of gene transcription, or that ERα acts as a transcriptional repressor by interfering with other transcription factors in its monomeric form [11]–[13]. In this context, it is important to state that another member of the steroid receptor family, the glucocorticoid receptor (GR), affects bone metabolism in its monomeric form independent of DNA-binding [14], whereas mechanistic insights into ERα signaling are still missing in regard to osteo-anabolic therapy.

In the current study, we have investigated the cellular and molecular mechanisms of the osteo-anabolic effect of pharmacological estrogen administration. For that, we implanted mice with subcutaneous estrogen pellets and analyzed their bone phenoype after 4 weeks of treatment. Our data show that estrogen treatment results in a FSH-independent increase in bone mass, which is mediated by the ERα in osteoblasts and primarily affects bone formation, not bone resorption. Furthermore, applying a mouse model carrying a mutation specifically disrupting DNA-binding of ERα, we demonstrate that the bone-anabolic effects of estrogen administration rely on this function of the ERα *in vivo*.

## Results

### Estrogen Administration at Pharmacological Doses Causes a FSH-independent Bone-Anabolic Effect

**Figure 1:**
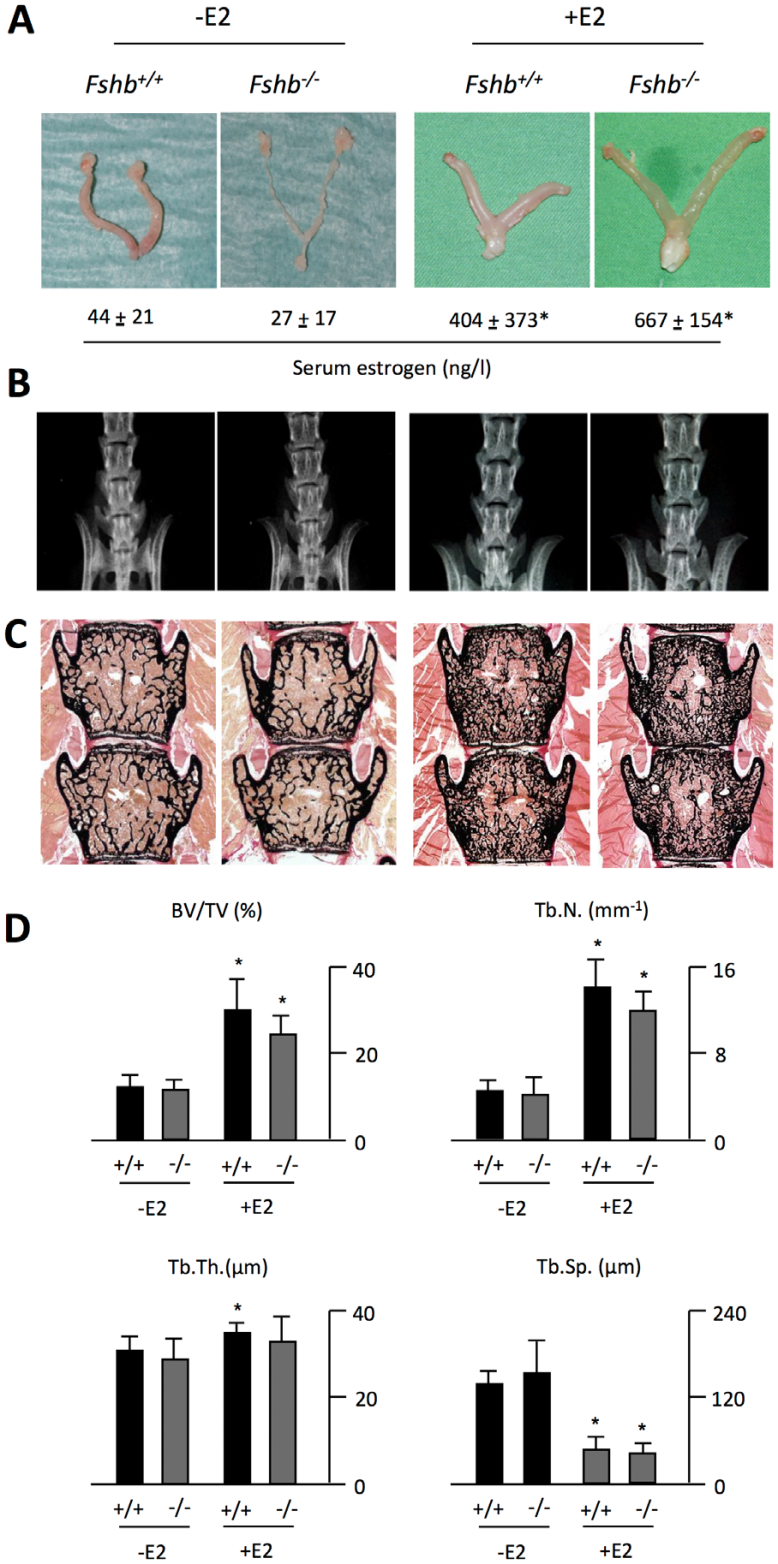
Estrogen administration causes an FSH-independent increase in vertebral bone mass. **A)** Gross anatomy of uteri and serum levels of estrogen in *Fshb^+/+^* and *Fshb^−/−^* mice 4 weeks after sham operation (−E2) or subcutaneous implantation of an estrogen pellet (+E2). **B)** Representative contact radiographs of the lumbar spine of the respective groups. **C)** Von Kossa/van Gieson-staining of non-decalcified spine sections and **D)** quantification of the trabecular bone volume per tissue volume (BV/TV), trabecular numbers (Tb.N), trabecular thickness (Tb.Th), and trabecular spacing (Tb.Sp). All data represent mean ± SD from at least 5 mice per group. *p<0.05 versus untreated control of each genotype.

To analyze whether the pharmacological effect of estrogen is dependent on FSH, we treated 12 week old wildtype and *Fshb^−/−^* mice with estrogen for 4 weeks. As expected, *Fshb^−/−^* mice displayed a marked atrophy of the uterus and ovaries, which was fully normalized by estrogen treatment (Fig. 1A). Serum measurements revealed that estrogen levels were non-significantly decreased in *Fshb^−/−^* mice, but significantly increased approximately 10-fold in the both groups following estrogen treatment (Fig. 1A).

Contact radiography of the lumbar spine showed an increase in bone mass in wildtype and *Fshb^−/−^* mice receiving estrogen (Fig. 1B). Confirming previous observations [8], non-decalcified histology and static histomorphometry of spine sections revealed normal trabecular bone volume in untreated *Fshb^−/−^* mice, despite their hypogonadism (Fig. 1C). Likewise, no significant difference in trabecular thickness, number and spacing between untreated wildtype and *Fshb^−/−^* mice was observed (Fig. 1D). Importantly, both wiltype and *Fshb^−/−^* mice receiving estrogen treatment displayed an approximately two-fold increase in the trabecular bone volume, which was explained by a striking elevation in trabecular numbers and a reduction in trabecular spacing (Fig. 1D).

**Figure 2:**
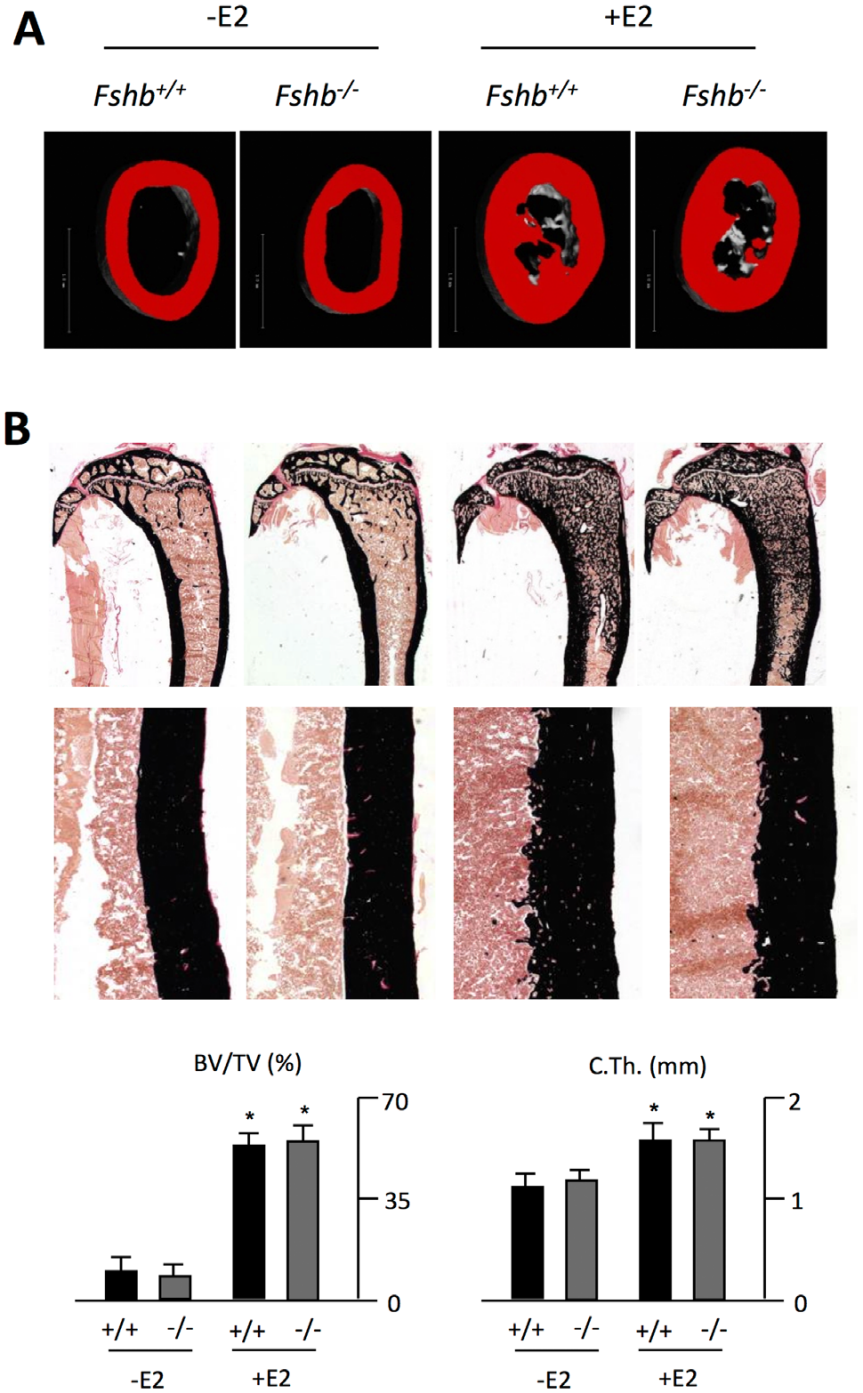
Estrogen administration increases bone mass in long bones independent of FSH. **A)** Representative cross-sectional µCT scans of the femoral diaphyses of untreated (-E2) or treated (+E2) *Fshb^+/+^* and *Fshb^−/−^* mice. **B)** Von Kossa/van Gieson-staining of non-decalcified tibia sections and quantification of the BV/TV and cortical thickness (C.Th.). All data represent mean ± SD from at least 5 mice per group. *p<0.05 versus untreated control of each genotype.

To evaluate whether similiar phenotypic differences are observed in long bones, we performed cross-sectional µCT scanning of the femur. Again, estrogen treatment did not only increase bone mass in the femur of wildtype, but also *Fshb^−/−^* mice (Fig. 2A). Non-decalcified histology confirmed these results, evidenced by a significant increase in both trabecular bone volume and cortical thickness in tibiae of wildtype and *Fshb^−/−^* mice receiving estrogen treatment (Fig. 2B).

### Pharmacological Estrogen Adminstration Enhances Osteoblast Function

**Figure 3:**
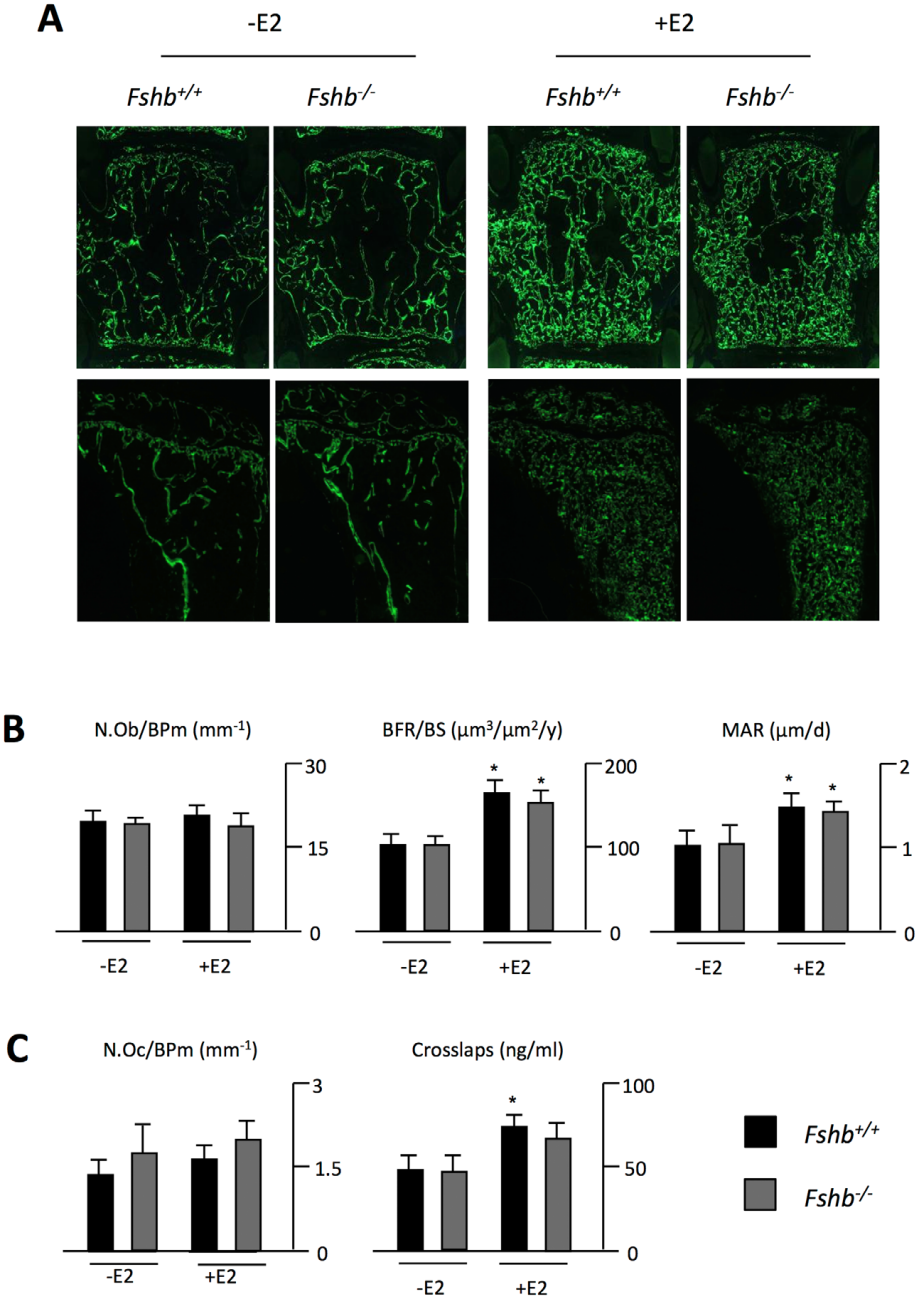
Estrogen treatment at pharmacologic doses increases osteoblast function independent of FSH. **A)** Fluorescent micrographs of non-decalcified spine (top) and tibia sections (bottom) of *Fshb^+/+^* and *Fshb^−/−^* mice 4 weeks after sham operation (−E2) or implantation of an estrogen pellet (+E2). **B)** Quantification of number of osteoblasts per bone perimeter (N.Ob/BPm), bone formation rate per bone surface (BFR/BS) and mineral apposition rate (MAR) in *Fshb^+/+^* and *Fshb^−/−^* mice. **C)** Quantification of number of osteoclasts per bone perimeter (N.Oc/BPm) and serum levels of collagen degradation products (Crosslaps) in the same groups. All data represent mean ± SD from at least 5 mice per group. *p<0.05 versus untreated control of each genotype.

To understand the nature of the observed effects, we performed cellular and dynamic histomorphometry of wildtype and *Fshb^−/−^* mice receiving no treatment or estrogen administration. Dual calcein injections revealed that estrogen treatment led to a marked increase of labeled surfaces and a greater distance between the labeling fronts in the vertebrae of both wildtype and *Fshb^−/−^* mice (Fig. 3A). Likewise, estrogen treatment resulted in a FSH-independent enhancement of calcein labeling in the tibia. Despite no alterations in osteoblast numbers in any group, both, the bone formation rate and the mineral apposition rate were markedly increased by estrogen treatment in wildtype and *Fshb^−/−^* mice (Fig. 3B). Increased amounts of collagen degradation products were measured in estrogen-treated mice, but assessement of bone resorption parameters failed to detect the expected change in osteoclast numbers (Fig.3C). Following adjustment of serum crosslaps for the increase in bone mass however, estrogen-treated animals displayed no net increase in relative bone resorption, implying that the rate of bone resorption is not affected by estrogen administration at pharmacological doses.

### The Osteoananabolic Effect of Estrogen Administration is Mediated by the ERα in Osteoblasts and Requires its DNA-binding

**Figure 4:**
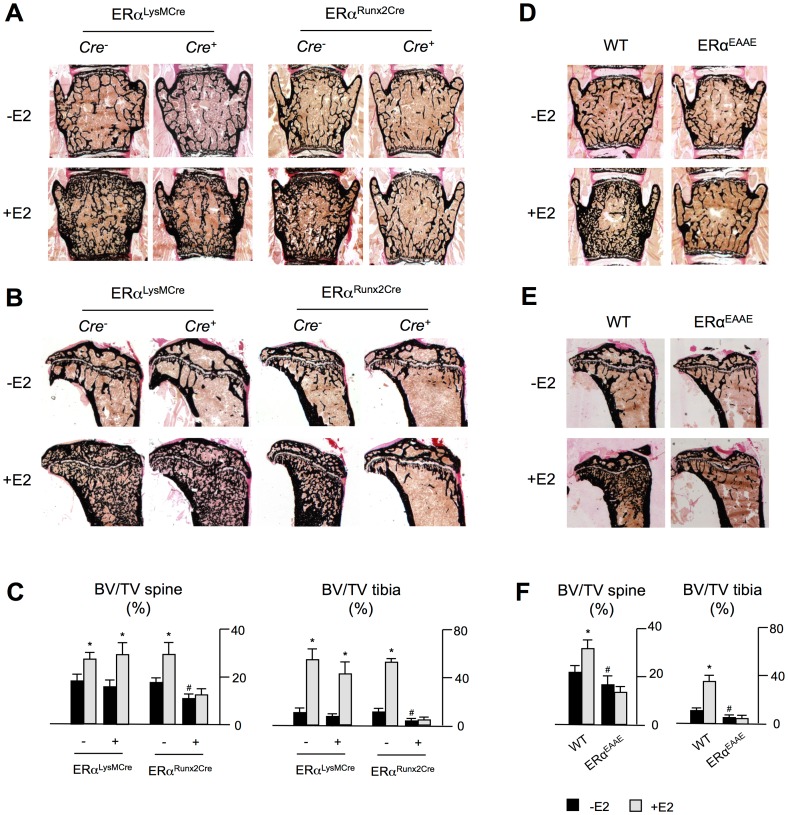
The osteo-anabolic effect of estrogen administration requires DNA-binding of ERα in osteoblasts. Von Kossa/van Gieson-staining of non-decalcified spine **A)** and tibia **B)** sections of *ERα^fl/fl^* mice carrying the *LysM-Cre* or the *Runx2-Cre* transgene, receiving the same treatment. **C)** Quantification of BV/TV in ERα^LysMCre^ and ERα^Runx2Cre^ mice receiving no treatment or estrogen treatment. Von Kossa/van Gieson-staining of non-decalcified spine **D)** and tibia **E)** sections of ERa^EAAE^ mice receiving no (−E2) or estrogen (+E2) treatment. **F)** Quantification of BV/TV in ERa^EAAE^ mice receiving no treatment or estrogen treatment. All data represent mean ± SD from at least 4 mice per group. ^#^p<0.05 versus control under basal conditions; *p<0.05 versus control following estrogen treatment.

In the next set of experiments we addressed the question, which cell type is primarily responsible for the observed effect of estrogen adminsitration. For that, we took advantage of mice carrying loxP sites 5′ and 3′ to exon 3 of *Nr3a1* gene encoding ERα (ERα^fl/fl^) [15]. These were crossed with *LysM-Cre* [16] or *Runx2-Cre* [14] mice to obtain specific deletion of ERα in osteoclasts and osteoblasts, respectively. Compared to *Cre* negative ERα^fl/fl^ controls, ERα^LysMCre^ showed no changes in the trabecular bone volume, whereas ERα^Runx2Cre^ displayed significantly decreased bone mass in spine and tibia sections relative to controls (Figure 4 A–C). Most importantly however, estrogen treatment of ERα^LysMCre^ mice led to a marked increase of the trabecular bone volume in the spine and the tibia, while this effect was completely abolished in mice lacking ERα in osteoblasts.

We finally addressed the question, whether the osteo-anablic effect of pharmacological estradiol administration requires the DNA-binding activity of ERα. For that purpose we took advantage of ERα^EAAE^ mice, which carry an ERα with an exchange of the amino acid residues Y201E, K210A, K214A and R215E in the DNA binding domain of the receptor. This mutant ER is not able to bind estrogen response elements (ERE) in the promoters of target genes, but the ERα protein is still recruited to AP-1 binding sites [17]. Compared to their littermate controls ERα^EAAE^ mice displayed a reduced trabecular bone volume in spine and tibia sections (Fig. 4 D–F). Most importantly however, the osteo-anabolic effect of pharmacological estradiol administration was completely abolished in these mice, thereby demonstrating that this effect requires the DNA-binding activity of ERα.

## Discussion

Although it is well established that sex steroids are powerful systemic regulators of bone remodeling [18]–[20], it is surprising that the cellular and molecular mechanisms of estrogen action on bone cells are still not fully clarified [3], [21], [22]. While it is generally accepted that the physiological function of estrogen is an inhibition of bone resorption, it remained questionable if this can solely explain the bone-anabolic effects of estrogen administration at pharmacological doses [23].

To understand the effects of pharmacologically administered estrogen, we first implanted subcutaneous pellets of estrogen into wildtype and *Fshb^−/−^* mice. Confirming previous reports, *Fshb^−/−^* mice displayed normal bone mass under basal conditions [8]. Most importantly however, estrogen administration caused an increase in bone mass in both wildtype and *Fshb^−/−^* mice, thereby demonstrating that pharmacological estrogen administration exerts a FSH-independent bone-anabolic effect. Surprisingly, we found that estrogen treatment primarily increased bone formation parameters, while we failed to detect the expected inhibitory effect on bone resorption. In this regard, it is important to state that we used relatively young mice at the age of 12 weeks, exhibiting intact gonads and no accelerated bone resorption due to estrogen deficiency. Moreover, although hypogonadic with dread-like utery and impaired folliculogenesis, *Fshb^−/−^* mice were still capable of producing estrogen and therefore do not resemble postmenopausal estrogen-deficiency in humans [24]. While this may explain the absence of an inhibitory effect of estrogen administration on bone resorption in our study, the increase in bone formation and mineral apposition rate, accounting for the striking osteo-anabolic effect, was rather unexpected.

Few studies so far have investigated the influence of estrogen treatment on osteoblast function *in vivo*. For example, it was found that, in addition to its anti-resorptive effect, estrogen specifically enhances bone formation in both healthy and ovariectomized rats [25]–[27]. Likewise, Nakamura et al. detected a significant increase in mineral apposition rate in ovariectomized mice implanted with estrogen pellets [5]. The observation that estrogen affects osteoblast function has also been demonstrated *in vitro*, however conflicting results have been reported. While several studies found no alteration [28] or even a decrease [29] in cell proliferation, Scheven et al. reported an increased proliferation of osteoblasts following stimulation with estrogen *in vitro* [30]. Likewise, estrogen was reported to differentially affect osteoblast differentiation and apoptosis, depending of the stage of cell maturation [31]. Furthermore, while estrogen was demonstrated to have either no effect or positively influence extracellular matrix production by cultured osteoblasts [32], experiments in our lab showed an inhibitory effect of long-term estrogen treatment on matrix mineralization (data not shown). These contradictory results may be explained by several reasons, including i) the application of highly variable immortalized cell lines and *ex vivo* culture systems of different origin and cell composition, ii) the different doses and timing of estrogen treatment in these cultures and iii) the lack of bi- or multidirectional communication between osteoblasts and osteoclasts and other cell types in most culture systems, potentially omitting cellular mediators important for estrogen action *in vivo* [33].

Given the striking osteo-anabolic effect of pharmacological estrogen administration observed *in vivo*, we thus chose to employ genetically modified mouse models to elucidate the underlying cellular mechanism. Applying transgenic mice expressing *Cre* recombinase under the control of the *LysM* or *Runx2* promotor, respectively, two previously well characterized and specific deleter strains [14], [16], we targeted ERα in osteoblasts and cells of the osteoclast lineage. Under basal conditions, ERα^Runx2Cre^ mice displayed a significantly decreased trabecular bone mass in the spine and tibiae. Most importantly however, the increase in bone mass following estrogen administration, which we observed in the corresponding control groups, was completely abolished in ERα^Runx2Cre^ mice. These data clearly demonstrate that the ERα in osteoblasts is the responsible mediator of the osteo-anabolic effect caused by pharmacological estrogen administration.

We further addressed the question, whether intact DNA-binding of ERα is required for the osteo-anabolic effect of pharmacological estrogen administration. Although ERα is a nuclear receptor mediating most of its pleiotrophic effects as a DNA-binding transcription factor, several reports have suggested that estrogen receptors are also capable of associating with the cell membrane to conduct rapid responses independent of gene expression [11], [12]. Alternatively ERα was reported to suppress gene transcription by interfering with DNA-bound transcription factors such as AP-1 [13]. In this regard it is important to state that we have previously reported that glucocorticoid-induced bone loss still occurs in mice carrying a glucocorticoid receptor incapable of DNA-binding [14]. Based on these findings, it was indeed relevant to use the ERα^EAAE^ mouse model in order to evaluate, whether estrogen-induced bone formation would also be independent of DNA-binding. Here we found that ERα^EAAE^ mice did not respond to the osteo-anabolic effect of estrogen administration. Therefore, although monomeric ERα signaling may be of relevance in some tissues [34], our data demonstrate that DNA-binding of ERα is required for pharmacologic effects of estrogen on bone mass.

Of interest, the applied treatment regimen in this study positively affected bone volume in both spine and long bones. This is clinically relevant, since fractures of the femur are associated with higher mortality than vertebral fractures [35]. At this point, the only FDA-approved osteo-anabolic substance for the treatment of bone loss disorders is Teriparatide, which requires daily injections for its beneficial action [36]. However, given the superior possibilities of estrogen delivery (e.g. orally, long-lasting patch) and its robust osteo-anabolic effect observed in this study, it may be acceptable to reconsider a temporary bone mass enhancement by estrogen treatment in selected postmenopausal women at high risk for osteoporotic fractures. Larger studies are required to evaluate if a relatively short-term treatment would outweigh the adverse effects of estrogen and SERM administration, such as an increased risk for uterine malignancies and venous thromboembolic events [37], [38].

## Materials and Methods

### Mice

*Fshb^+/−^* mice were obtained from the Jackson Laboratory (# 003283) and genotyping was performed as described previously [24]. Due to the infertility of *Fshb^−/−^* females, we only used wildtype and *Fshb^−/−^* littermates derived from *Fshb^+/−^* matings for our analysis. The generation and genotyping of ERα^fl/fl^ and ERa^EAAE^ mice has been described previously [15], [17]. The local authorities of Hamburg, Germany (Free and Hanseatic City of Hamburg, Germany; Public health authority; Veterinary Office; File reference: G8151/591-00.33, No. 16/2000) approved all experiments.

### Surgical Procedure

For estrogen treatment 12 week old female mice of each genotype were implanted with an estrogen pellet (Innovative Research of America, Sarasota, FL, with a release rate of 0.36 µg/day) in the scapular region behind the neck. Due to the preantral stage block in folliculogenesis in female *Fshb^−/−^* mice, synchronization of the reproductive status was not performed prior to surgery. To assess dynamic histomorphometric indices estrogen-treated and sham-operated mice were given two injections of calcein 9 days and 2 days before sacrifice. Radiographic and histologic analyses were performed after a treatment period of 4 weeks. To evaluate the impact of estrogen treatment on the gonads, the size of the ovaries was monitored immediately after sacrifice.

### Radiographic and µCT Analyses

After removal of internal organs, the whole skeletons were fixed in formalin as described previously [39]. Skeletons were subsequently analyzed by contact radiography using a Faxitron X-ray cabinet (Faxitron X-ray Corp., Wheeling, IL, USA). To determine the cortical thickness, cross-sectional scans of the femur were performed using a µCT 40 (ScancoMedical, Bassersdorf, Switzerland) at a resolution of 10 µm. The raw data were manually segmented and analyzed with the µCT Evaluation Program V4.4A (Scanco Medical). For visualization, the segmented data were imported and displayed in µCT Ray V3.0 (Scanco Medical).

### Non-decalcified Histology

After an incubation in 70% ethanol for 24 hours, the lumbar vertebral bodies L2 to L5 and one tibia of each mouse were dehydrated in ascending alcohol concentrations and embedded in methylmethacrylate as described previously [40]. Sections of 4 µm thickness were cut in the saggital plane on a Microtec rotation microtome (Techno-Med, Munich, Germany). These sections were stained with toluidine blue and by the von Kossa/van Gieson procedure as described [40]. Nonstained sections of 12 µm were used to determine bone formation rate.

### Histomorphometry

Histomorphometric quantification was performed on toluidine blue–stained sections. Analysis of the trabecular bone volume (BV/TV), trabecular thickness (TbTh), trabecular number (TbN), trabecular spacing (TbSp), and the determination of osteoblast and osteoclast numbers (Ob.N/BPm; Oc.N/BPm) were carried out using the Osteo-Measure histomorphometry system (Osteometrics, Atlanta,GA, USA) according to the guidelines of the American Society for Bone and Mineral Research [41]. The bone formation rate and mineral apposition rate was determined by fluorescence microscopy on 12 µm thick sections.

### Serum Analysis

Estrogen levels were determined by the Exciting Systems (Roche/Hitachi Modular Analytics Hybrid; Roche Diagnostics, Mannheim, Germany) at the Institute of Clinical Chemistry, University Medical Center Hamburg-Eppendorf, Hamburg, Germany. To evaluate the rate of bone resorption we quantified the serum concentrations of collagen degradation products (Crosslaps) by ELISA (Immunodiagnostic systems, #AC-06F1).

### Statistics

All data are presented as mean+SD. Statistical analyses of the results were performed using two-way-ANOVA (IBM SPSS Statistics 20). Statistical differences were considered significant when p<0.05.

## Acknowlegments
